# The Science and Art of Surgery

**Published:** 1885-07

**Authors:** 


The Science and Art of Surgery. A Treatise on Surgical Injuries, Diseases and Operations. By John Eric Erichsen, F. R. S., L. L. D., F. R. C. S., Professor of Surgery and of Clinical Surgery in University College, etc. Eighth edition. Revised and edited by Marcus Beck, M. S., F. R. C. S., Surgeon to University College Hospital. Philadelphia: H. C. Lea's Son & Co. 1885.

Erichsen's surgery needs no introduction from us. It has been a standard work in this country, as well as in Great Britain, for more than thirty years, and has steadily grown in favor with both practitioners and students. The last edition is deserving of all praise. In its preparation, the distinguished author has called to his aid the services of distinguished specialists. All that was antiquated has been dropped from its pages, and the most recent advances in modern surgery have been incorporated in the work. The first volume contains 1124 pages, the second over 1200; although we cannot, in the limited space at our command, refer to the excellence of individual chapters, we are compelled to say that a rather close examination has failed to show us even a chapter where we could criticise save in that on "Injuries of the Spine." The views advanced in his well-known work on "Concussion of the Spine" are here restated and will not be unanimously agreed with. As a whole, however, "Erichsen's Surgery" is unsurpassed, and the present edition is more than ever worthy of professional favor.

